# A Case of Acquired Cystic Kidney Disease From Chronic Renal Failure Without Dialysis

**DOI:** 10.7759/cureus.70396

**Published:** 2024-09-28

**Authors:** Barbara Chiu, Isabel Diaz, Emily Quintero, Kebir Bedran

**Affiliations:** 1 Radiology, Florida International University, Herbert Wertheim College of Medicine, Miami, USA; 2 Medicine, Florida International University, Herbert Wertheim College of Medicine, Miami, USA; 3 Family Medicine, Baptist Health South Florida, Miami, USA; 4 Internal Medicine, Baptist Health South Florida, Miami, USA

**Keywords:** acquired cystic kidney disease (ackd), chronic kidney disease, chronic renal failure, polycystic kidney, renal cyst

## Abstract

The kidneys play a vital role in maintaining homeostasis by filtering the blood of waste products and toxins, maintaining a delicate balance of fluids and electrolytes, and closely regulating blood pressure via the juxtaglomerular apparatus. Cystic kidney disease is a pathology of the kidneys that can be either acquired or congenital. This case report introduces a seventy-eight-year-old male with a medical history of a myocardial infarction and heart valve replacements who presented to the emergency department due to a recent fall at home unrelated to syncope. As he rose from his chair, the patient fell forward but was able to pick himself up from the ground. Upon further review, lab work revealed end-stage renal disease with a creatinine of 19.7 mg/dL (reference range: 0.6-1.2 mg/dL) and blood urea nitrogen of 186 mg/dL (reference range: 7-18 mg/dL). Potassium was 5.2 mmol/L (reference range: 3.5-5.1 mmol/L) and the electrocardiogram showed normal sinus rhythm. Urinalysis showed proteinuria of 100 mg/dL with high white blood cells 28/High-power field and red blood cells (RBCs) 3/HPF but no bacteria. Trace blood and leukocyte esterase were also noted. Microscopic analysis was significant only for epithelial cells 1/HPF. The patient also had symptomatic anemia with a hemoglobin of 6.4 g/dL, which contributed to his symptoms on presentation. Computed tomography of the abdomen and pelvis without intravenous or per oral contrast revealed numerous bilateral renal parenchymal cysts. After receiving two units of packed RBCs for severe anemia, he underwent several dialysis treatments during his stay. The patient denied a family history of polycystic kidney disease, though he had a deceased sister with unspecified “kidney problems.” Acquired cystic kidney disease (ACKD) before dialysis is a rare phenomenon that prompts a careful history, physical exam, and diagnostic evaluation to avoid misdiagnosis and improper treatment. Although rare and not cited often in the literature, ACKD should be entertained in the differential diagnosis of this presentation.

## Introduction

Acquired cystic kidney disease (ACKD) is defined as the presence of three or more renal cysts bilaterally on imaging without hereditary cause [1]. It is most often associated with long-term dialysis, with approximately 90 percent of patients on dialysis for eight years developing ACKD [2]. However, there are some reports of ACKD developing in patients with chronic renal insufficiency but who were not yet dialyzed [3]. The pathogenesis of this disease is still not fully understood but several precipitating factors have been proposed. These include obstruction of the proximal tubules by calcium oxalate crystals and interstitial fibrosis, renal ischemia-reperfusion injury triggering cystogenesis signaling pathways, and over-expression of proto-oncogenes (such as c-Jun) and epidermal/hepatocyte growth factors causing tubular epithelium hypertrophy [4,5]. It is thought that the declining renal function and nephron loss with chronic kidney disease may excessively trigger the compensatory hypertrophy mechanisms listed above [6].

ACKD can present similarly to autosomal dominant polycystic kidney disease (ADPKD); however, some clinical features differ between the two diseases. One important distinction is the hereditary component of ADPKD; patients often have a family history of several affected relatives, whereas ACKD is a direct result of uremic injury to the kidney. Furthermore, the kidneys are generally not as enlarged as in ADPKD and there is no extrarenal involvement (such as berry aneurysms or hepatic cysts) [5]. 

Presentations of ACKD from end-stage renal disease alone (without starting dialysis) have not been widely reported within the literature, as indicated by three case reports of ACKD from end-stage renal disease [7]. Here, we report the case of a seventy-eight-year-old male who was incidentally diagnosed with ACKD from years of untreated end-stage renal disease. In light of this, it is imperative that practitioners become aware of the possibility of ACKD even before dialysis is started and understand the potential complications of this disease.

## Case presentation

The patient is a seventy-eight-year-old male with a past medical history of a myocardial infarction four years ago who presented to the emergency department due to a recent fall while getting up from a chair at home. He had bilateral leg pain on arrival but denied head injury, loss of consciousness, chest pain, or palpitations. Further history-taking revealed decreased appetite, generalized weakness, fatigue, pruritus, and dizziness when getting up from a chair, which had lasted over a month. He denied orthopnea, nocturnal paroxysmal dyspnea, and exertional angina or dyspnea, which ruled out acute congestive heart failure from our differential.

The patient also had a coronary artery bypass graft (CABG) procedure done four years ago with three heart valve replacements five years ago; his ejection fraction was 45 percent at the time. After the procedure, he was able to walk a few blocks without shortness of breath or chest pain and was able to perform all activities of daily living independently. Upon further history gathering, the patient states that he did not regularly visit the doctor and that he came to the hospital three to four years ago for dizziness, where his creatinine was found to be 3.5 mg/dL (reference range: 0.6-1.2 mg/dL). He was advised to follow up with a nephrologist but never did. He also mentioned that his sister had passed away with unspecified “kidney problems” but denied any other family history of renal failure and dialysis. No renal ultrasound was done at the time as numerous cysts on previous abdominal and pelvic CT were reported.

**Figure 1:**
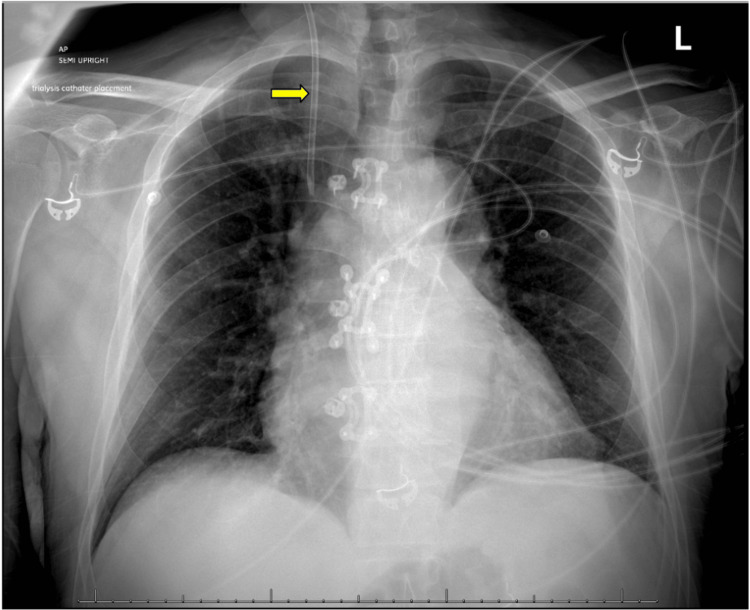
Chest X-ray showing normal heart size without consolidations or pleural effusion and a right internal jugular catheter indicated by the arrow

**Figure 2:**
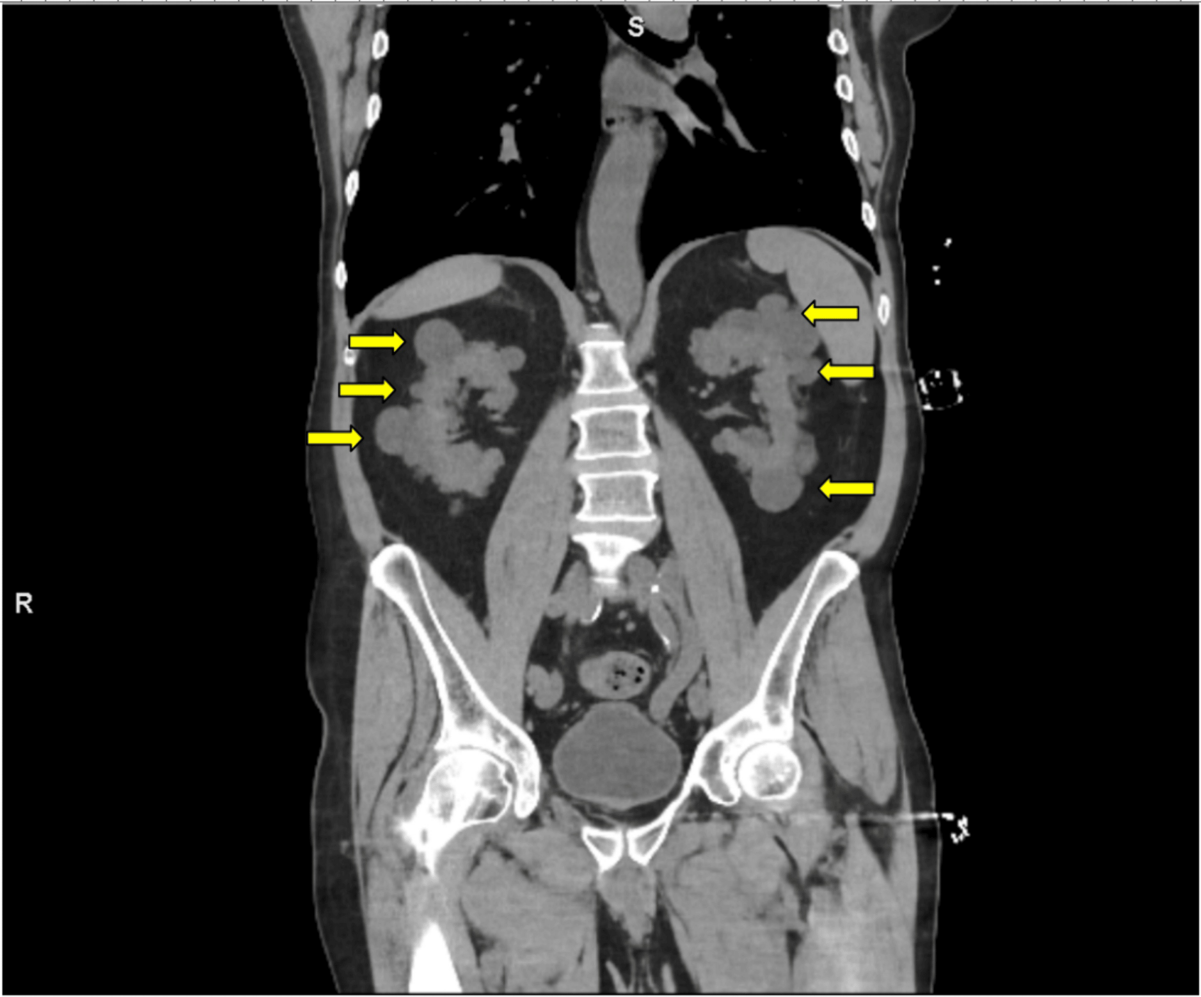
Coronal abdominal and pelvic CT scan W/O contrast showing numerous bilateral renal parenchymal cysts indicated by arrows

On arrival, the patient's vitals were as follows: temperature 37.0 C, heart rate 80 beats/min, respiratory rate 14 breaths/min, blood pressure 151/78, and O2 saturation 98%, with a body mass index of 25.97. The patient was alert and in no acute distress; physical exam revealed normal (not distant) heart sounds, no rub, appropriate mental status, no edema of the lower extremities, no jugular vein distention, and no uremic frost on skin examination. The rest of the physical exam was non-revealing. His laboratory studies revealed a hemoglobin of 6.4 g/dL (reference range: 13.5-17.5 g/dL), creatinine of 19.7 mg/dL (reference range: 0.6-1.2 mg/dL), blood urea nitrogen (BUN) of 186 mg/dL (reference range: 7-18 mg/dL), potassium of 5.29 mEQ/L (reference range: 3.5-5 mEQ/L), and a high anion gap (16 mmol/L, reference range: 2-15 mmol/L) metabolic acidosis with a CO2 of 8 mm Hg (reference range: 33-45 mm Hg). Parathyroid hormone was found to be 787.60 pg/ml (reference range: 6-96 pg/mL). Urinalysis showed 100 mg/dL protein with high RBCs (3/HPF) and WBCs (28/HPF) but no bacteria. Trace blood and leukocyte esterase were also noted. Microscopic analysis discovered 1/HPF epithelial cells. The patient also had symptomatic anemia with a hemoglobin of 6.4 g/dL, which contributed to his symptoms on presentation. X-ray of the left and right femur and knee, bilateral hip, and pelvis showed no fractures. A chest x-ray also showed evidence of prior sternotomy, CABG, and right internal jugular central line catheter; heart size was within normal limits and no consolidation or pleural effusion was seen (Figure 1). Abdomen and pelvic computed tomography (CT) scan without IV or PO contrast showed multiple cysts on both kidneys (Figure 2), diverticulosis without acute diverticulitis, and nonobstructive left intrarenal calculi. Brain CT without contrast had no findings of hydrocephalus, acute intracranial hemorrhage, extra-axial fluid collections, or lobar infarctions.

The patient was admitted on April 30th due to acute renal failure with high anion gap metabolic acidosis and severe anemia secondary to progression of chronic kidney disease (CKD). He was emergently started on dialysis through a right internal jugular vein temporary hemodialysis catheter. Gastroenterology was consulted as the patient had reported previous episodes of melena but stated that no emergent procedure was necessary at the time; the patient was not iron deficient (iron 19.7 ng/mL, total iron-binding capacity 77 mcg/dL) suggesting his anemia was likely from advanced CKD. One unit of packed red blood cells was transfused, followed by another unit on day two of the admission. 

On day seven of the admission, the patient was sent to vascular surgery for arteriovenous fistula creation and to place a tunneled dialysis catheter. The patient reported right-sided chest pain described as “pressure” but he denied left arm pain, radiating pain, and jaw tightness. The patient had atypical chest pain, with his troponins negative x2 (33 and 66 ng/L) and his electrocardiogram demonstrated normal sinus rhythm with left axis deviation, nonspecific ST-T wave abnormalities, and occasional premature ventricular complexes. His pain resolved after a single dose of morphine and the patient had no more episodes of chest pain throughout his hospital stay. 

The 2D echocardiogram showed an ejection fraction of 45% with paradoxical left interventricular septal motion, regional wall motion abnormalities, and grade I diastolic dysfunction. Mild aortic regurgitation was present, but all valves were otherwise normal in structure and function. No significant pericardial effusion present. Cardiology was consulted and determined that chest pain was likely due to recent catheter placement and severe anemia and recommended to continue aspirin and statin. The patient was unable to tolerate beta blocker due to hypotension. More invasive cardiac testing was not done due to severe anemia and thrombocytopenia.

The patient was discharged home with recommendations to follow up with a nephrologist and dialysis was arranged Monday, Wednesday, and Friday (MWF). On discharge, his hemoglobin went up to 8.7 g/dL (reference range: 13.5-17.5 g/dL), creatinine 8.62 mg/dL (reference range: 0.6-1.2 mg/dL), and BUN 53 mg/dL (reference range: 7-18 mg/dL), and his estimated glomerular filtration rate (eGFR) was 7.29 mL/min/1.73m2. Considering these findings and the patient’s clinical presentation, he was diagnosed with ACKD from years of end-stage renal disease as the patient had significantly decreased renal function but was asymptomatic until this hospital visit. Post-discharge, the patient is tolerating inpatient MWF dialysis well with no overload and normal electrolyte levels. He is currently taking phosphorus binders and calcitriol for CKD mineral bone disease, and epoetin alfa for his renal anemia.

## Discussion

ACKD is often asymptomatic and diagnosed incidentally on imaging with the presence of three or more cysts in each kidney [1]. However, patients also commonly present with urinary tract infections, nephrolithiasis, flank pain, and hematuria [8]. The renal cysts in ACKD have potential to rupture and cause mild to severe bleeding. While mild bleeding can be treated with analgesics to manage symptomatic pain, heavier bleeds that do not resolve spontaneously indicate the need for renal embolization or potential nephrectomy [9]. Another serious complication is an increased risk (nearly hundred-fold) of developing renal cell carcinoma. ACKD is considered to be a premalignant state as many of the proto-oncogenes and growth factors involved in its pathogenesis can also lead to cancer [10,11]. Though the renal cell carcinomas associated with ACKD tend to be small and not very aggressive, it is vitally important for clinicians to recognize patients with ACKD and closely follow up knowing their risk factors [5]. 

Currently, there is no one standardized protocol to screen for ACKD, although ultrasound has been suggested in at-risk populations (those with chronic kidney disease and/or who are on dialysis) to detect and follow up on cyst development [5]. CT is the most sensitive method of detection; however, it is more costly and exposes patients to greater radiation [3]. Treatment for ACKD usually involves several years of dialysis and targeted therapy for the side effects of CKD, such as phosphate binder therapy for mineral bone disorder and erythropoietin for renal anemia. The ultimate treatment is a renal allograft, which results in regression of the cystic nature of the disease and the return to its baseline atrophic size. However, transplanted renal allografts are not totally effective as the transplanted organ can become susceptible to the development of ACKD if prolonged rejection and renal failure occur [12].

## Conclusions

ACKD is a rare finding, even more so when it occurs before dialysis. This case demonstrates the importance of recognizing various presentations of ACKD and the importance of physicians being alert in at-risk populations, especially the elderly. Due to the possibility that ACKD can often be confused for autosomal dominant kidney disease, it is important to obtain a brain CT to look for cerebral berry aneurysms and to look for cysts in the liver, pancreas, spleen, ovary, and testicles. Despite our patient’s low creatinine and eGFR values, CT of the abdomen and pelvis without intravenous contrast showed no renal cell carcinoma. Fortunately, the patient came to the hospital to seek care for his syncope and incidentally was able to obtain diagnosis that explained the symptoms that he presented with. It is our hope now that the patient attends his follow-up appointments and remains compliant with his care, especially his dialysis appointments.
